# Young Adults’ Use of Different Social Media Platforms for Health Information: Insights From Web-Based Conversations

**DOI:** 10.2196/23656

**Published:** 2022-01-18

**Authors:** Megan S C Lim, Annika Molenaar, Linda Brennan, Mike Reid, Tracy McCaffrey

**Affiliations:** 1 Burnet Institute Melbourne Australia; 2 Monash University Melbourne Australia; 3 University of Melbourne Melbourne Australia; 4 RMIT University Melbourne Australia

**Keywords:** social media, Facebook, Instagram, YouTube, health information, health communication, young adults

## Abstract

**Background:**

Social media–delivered health promotion has demonstrated limited uptake and effectiveness among young adults. Understanding how young adults interact with existing social media platforms for health might provide insight for future health promotion interventions.

**Objective:**

The aim of this study is to describe how young adults interact with different social media platforms for health and health information.

**Methods:**

We used a web-based conversation methodology to collect data from 165 young adults aged 18 to 24 years. Participants participated in an extended conversation with moderators and other participants about health and social media. They were prompted to discuss how they find health information, how they use different social media platforms, and how they evaluate the trustworthiness of information. A thematic qualitative analysis was applied to the data.

**Results:**

Young adults spent a lot of time scrolling through Facebook newsfeeds, which often resulted in seeing health-related content either from their friends, news sources, or advertisements. Some actively sought out information about specific health areas by joining groups or following relevant pages. YouTube was considered a useful source for learning about everything and was often the go-to when searching for information or advice (after Google). Young adults found the video format easy to learn from. They stated that they could identify accurate YouTube health content by cross-checking multiple videos, by feeling that the presenter was *real* and relatable, or just through instinctively judging a video’s credibility. Instagram was a source of inspiration for health and wellness from those whose lives were dedicated to healthy lifestyles and fitness. Twitter, Tumblr, and Snapchat were rarely used for health information.

**Conclusions:**

Most young adults obtain health information from social media, both actively and through passive exposure. Participants indicated looking to social media influencers for health and lifestyle inspiration and judged the credibility of sources by appearance and instinct. Health experts should try to use the channels in the way that young adults already use them; use relatable role models on Instagram and YouTube, eye-catching headlines and support groups on Facebook, and easy to follow instruction videos via YouTube.

**International Registered Report Identifier (IRRID):**

RR2-10.1111/1747-0080.12448

## Introduction

### Health Promotion Via Social Media

In recent years, there has been a proliferation of social media–delivered health promotion campaigns targeting young adults. Such campaigns use the immense popularity of social media platforms to reach young adults with messaging to improve their health behaviors and outcomes. However, despite their promise, many interventions have demonstrated poor reach or limited effectiveness. For example, a systematic review found that only 1 of 9 interventions had a statistically significant positive impact on nutritional outcomes among young adults [1]. The proportion of young adults engaging with social media interventions in this review (eg, by liking, commenting on, or sending tweets) varied from 3% to 69%, and in another review, young adult participants engaged in only between 5% and 15% of the intended interactions [2]. The uptake and acceptability of health promotion on social media has been low among young people [1,3,4].

A systematic review showed that health promotion on social media primarily comprised information dissemination and providing social support [1]. Information dissemination can be achieved through posts from the organization that may reach the target group directly, if they have chosen to follow the organization, or an organization can pay to boost the reach of posts to others in the target group (paid advertising). Social support requires relevant individuals to interact with each other. Such social networks occur naturally (eg, in private groups on Facebook), but if health experts were to set up a group, they would need existing contact with target groups or paid social media advertising.

However, young people may not want to access health information or health social support via social media. The lack of success achieved by interventions may be based on a mismatch between how young adults use social media and the way health promoters use it. Research shows that most young people prefer to source health information from websites and not from social media [5,6]. Furthermore, many perceive it as socially undesirable to discuss personal health topics such as body weight in a public forum such as social media [1,4,7,8]. Consequently, the goals of the health promoter are not congruent with the functions of the social media platform from the users’ point of view.

### How Young People Use Social Media for Health

Furthermore, social media is not a single entity. There are many different social media sites or platforms, each with different features, target demographics, and use practices (affordances). Previous reviews have found that almost all health promotion interventions using social media described in the literature have been delivered through Facebook, Twitter, or custom-built social networking sites [1,3,9]. However, young adults are turning away from these platforms toward image- and video-based services such as Instagram, Snapchat, and Tik Tok [10-12]. Building a novel platform for a health intervention is costly and negates the major advantages of social media, which is that young people are already there in large numbers and are familiar with platforms’ appearance and functionality [9].

Understanding how young adults interact with existing social media platforms for health might provide insight for future health promotion interventions; however, few studies have made a distinction between the use of different platforms. For example, a survey of 396 college students from the United States found that information sharing was a major motivation for Facebook use, whereas Instagram and Snapchat were used more for self-expression and self-documentation [11]. Research with adolescent girls revealed that Snapchat was used for humor, Instagram was used for self-presentation, and YouTube was used for wasting time [13]. A survey of college students from the United States showed that Facebook and Snapchat were connected with real-life friends, whereas Twitter and Instagram were used to connect with strangers [14]. Ethnic Chinese adolescents living in the United States reported viewing pictures of food on Snapchat and Instagram, whereas Facebook was used more for sharing information about health and disease [15]. In 1 study, 12 women college students journaled their use of social media and described using Facebook for social support and information gathering, whereas Pinterest and Instagram were used for recipes, exercise regimens, and inspiration [16].

In addition, health information on social media platforms from health experts competes with health information from nonexperts and industry marketing [17]. Health promotion content on social media is created by health organizations and health professionals with the intention of improving the health and well-being of recipients. Fast food, alcohol, and other commercial companies are very successful in reaching young adults on social media [18,19], as are individuals such as health and lifestyle influencers, wellness gurus, and fitspiration models [17,20,21]. In the social media era, health and science experts are often less highly regarded than celebrities and social media influencers for health and lifestyle advice [22,23]. The proliferation of health information on social media means it is difficult for laypeople to differentiate an evidence-based message from misinformation in an environment where everyone appears to have expertise [24,25]. A recent review found that the credibility of social media health information is affected by language used, perceived expertise, and bandwagon cues, such as the number of likes a post receives [26]. Most studies have examined Facebook and Twitter, whereas trustworthiness on Instagram and YouTube has been studied less frequently [26,27]. The research shows that credibility is judged differently on the 2 platforms; for example, personalized language was an effective way to increase credibility on Facebook, but depersonalized tweets were more credible on Twitter [27,28].

Although patterns emerge from these studies, there is a need for further understanding of how young adults use different platforms to seek out health information and whether they trust this information. This can be used to improve our delivery of health promotion on social media and help us understand how social media can support public health campaigns. We hypothesized that young adults would use different social media platforms in different ways and sought to determine what these differences were.

### Objective

The aim of this study is to describe how young adults interact with different social media platforms for health and health information.

## Methods

### Design

This study used qualitative data from phase 1a of the Communicating Health project [29]. The aim of the project was to understand the use of social media by young adults to engage with health-related information; improve the effectiveness of social media strategies to motivate, engage, and retain young adults in interventions to reduce the risk of obesity; and identify and disseminate effective ways to deliver these interventions via social media.

Phase 1a of Communicating Health used a web-based conversation methodology to collect data from young adults. This methodology can generate rich insights from participants using the principles of digital ethnography—a process of direct and sustained digital engagements with participants in the context of their daily lives [30]. Participants in web-based communities converse over time and respond to questions from group moderators. The conversations lasted 4 weeks and covered a series of social media–, health-, and eating-related topics. They were hosted and moderated by an independent marketing research agency and took place in a specifically created digital lounge room.

### Participant Recruitment

A research society–certified field house was used to recruit participants from 3 different International Organization for Standardization accredited panels [31,32]. Young adults who had previously consented to participate in the research with 1 of 3 market and social research panels were invited to participate in this study. Participants were also able to invite their friends who were then required to undergo the same screening and profiling process. Participants who contributed to all of the forums, challenges, and polls received a gift voucher for Aus $100 (US $71), and the 20 participants who gave the most exhaustive contributions received an additional remuneration of Aus $100 (US $71).

**Figure 1:**
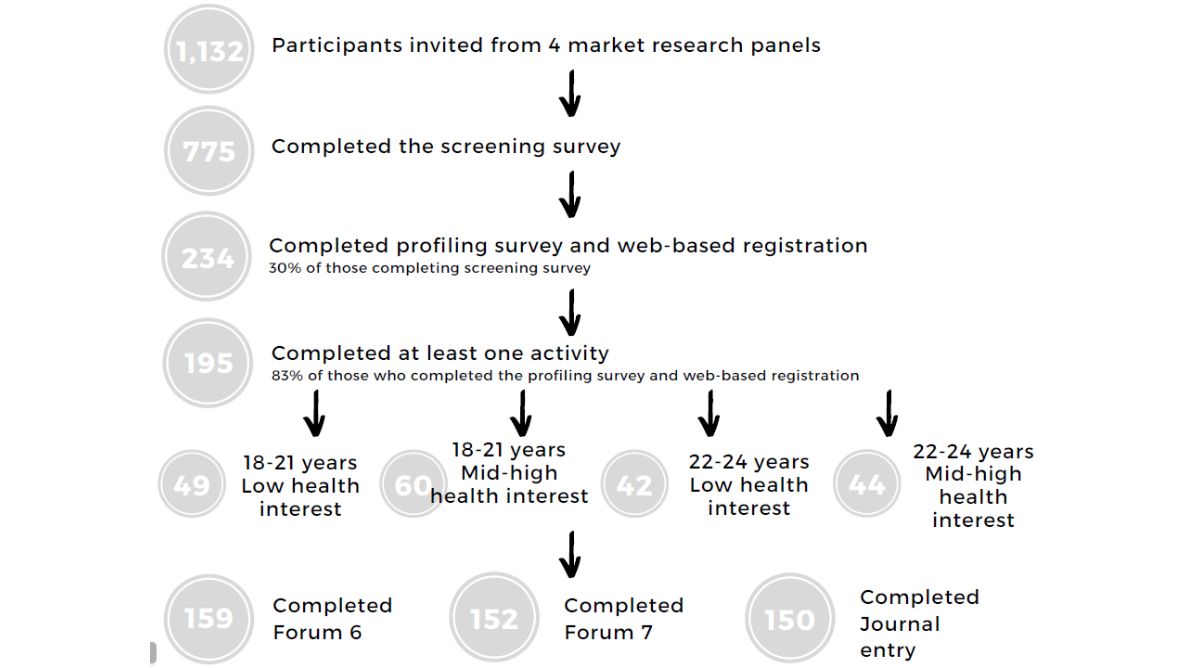
Participant flow through screening and research.

Figure 1 summarizes participant flow. Research panels invited participants by email to complete a screening survey (completed by 775 participants). Young adults (aged 18-24 years) using social media at least twice daily and living in Australia were eligible to participate. Eligible participants were then invited to register themselves on the digital lounge room used to host web-based conversations and complete a profiling survey. The profiling survey included self-reported weight and height, demographic information, social media use, and interest in health (completed by 234 participants). This was used to assign individuals to 4 approximately equal web-based communities based on age, 18-21 years and 22-24 years, and interest in health, low or mid to high. The classification of interest of health was based on the median split from the following question completed in the profiling survey: “On a scale of 1-7 where 1 means ‘Strongly Disagree’ and 7 means ‘Strongly Agree’, please indicate how strongly you agree with the following statement—'I take an active interest in my health’.” These groups were not analyzed separately but were used so that participants would be placed with those of more similar viewpoints. Finally, 195 people participated in at least one activity in the web-based conversations.

### Procedure

The web-based conversations (data collection) began on May 10, 2017, and the website remained active until June 6, 2017. Participants accessed the web-based community by logging in and creating a username. They were known to moderators and other participants only by this self-selected username. Participants then joined in an extended conversation about health and social media, in which they could respond to the insights of other participants and to prompts guided by the web-based moderators. Moderators asked for follow-up and clarification of participant responses and gave minimal prompts beyond the opening questions. The moderators included both a female moderator (Bachelor of Arts Psych Social and Master of Arts Applied Social Research) and a male moderator (Master of Management Marketing and Finance) with long-standing experience in market research.

There were 20 different forum discussions, in addition to 3 polls, 2 challenges, and 1 journal entry. For this manuscript, data in the form of text responses and uploaded images from the journal entry and 2 of the forums were analyzed (Textbox 1). These were chosen because of their specific discussion on the use of social media to source health information.

**Discussion prompts for discussion forums included in analysis.**

**Discussion prompts**

- Forum 6—my sources of health information
  “Now we’d like to know about the sources of information that we use in relation to finding out about health and healthy lifestyles, so post here anything that you have accessed/or access currently and in the process, tell us what’s so interesting about them. If you can’t think of any, that’s fine, just say so. If you come across some new ones in the course of this community, please post them here too.”
- Forum 7—seeking health information
  “Imagine you're looking to find out a bit more information about health (you choose which topic you want to research): where are you going? Please step us through your search: which pages you went to, how you came across them, what you found interesting, what you found frustrating...Did anything make you trust what you saw? How and why? What would make you distrust the source? (i.e. what sends the bullshit alarm ringing?) Please share any screenshots of the websites/social media pages/profiles, links to them and any other visuals you might have around your search!”
- Journal (my use of social media and internet today)
  “In this ‘journal’, we want you to record your daily use of social media and the internet at least 4 times across the three weeks of this community.”
  “Please list below the social media sites you have used, how often and for how long today (you can use yesterday if it’s still early in the day today). Don’t forget to tell us over the course of four different days!”
  “Did you notice any ad, product, company or brand...? Tell us more about what made you notice them...”
  “Beyond the everyday topics, what was discussed amongst your friends? E.g. my friends post about the politics of the day or about ads that are controversial or things like that...Did you engage in that conversation? How?”

### Analysis

Analysis was conducted using NVivo 11 (QSR International). Following data familiarization, analysis commenced with a deductive phase to test the hypothesis that young adults would seek and be exposed to health information differently on different web-based platforms. To do this, forum and journal data were sorted by the platform types that were mentioned to create multiple sets of data, specifically Facebook, YouTube, Instagram, Pinterest, Snapchat, Twitter, Tumblr, and other platforms. Both forums and all journal entries were grouped together based on the different platforms in this stage for collective interpretation. Then, each data set was analyzed separately using an inductive approach to identify themes relating to how each platform was used for health information. Text was coded manually, and codes were grouped into themes. Data and subthemes were presented grouped under each platform, and each platform was given a theme title to best convey its distinct attributes. Quotes were presented verbatim, with identifying information redacted, and may include spelling errors.

Investigator triangulation was used to enhance the rigor of the analysis where coding of all forum responses and development of initial themes was conducted independently by 2 authors before coming together to discuss and come to a consensus on final themes [33]. The authors who analyzed the data included MSCL, a public health researcher (Bachelor of Biomedical Science, PhD), and AM, a nutrition scientist (Bachelor of Nutrition Science).

Descriptive quantitative findings from the profiling survey relating to sociodemographic variables, social media use, sourcing health information on the web, and trust in health information on the web were also presented.

### Ethics and Funding

This study received ethics approval from the Royal Melbourne Institute of Technology Business College Human Ethics Advisory Network (project number 20489) and Monash University Human Research Ethics Committee (project number: 7807). The Communicating Health project was funded through an Australian National Health and Medical Research Council Targeted Call for Research into Engaging and Retaining Young Adults in Interventions to Improve Eating Behaviours and Health Outcomes (GNT1115496).

## Results

### Participant Characteristics and Social Media Use

A total of 165 participants engaged in at least one of the forums were included in this analysis. Owing to the forums and journal entry being released in different weeks, there were different completion rates for each (forum 6: 159/165, 96.4%; forum 7: 152/165, 92.1%; and journal entry: 150/165, 90.9%). Participant characteristics for those who completed at least one of these activities is shown in Table 1.

**Figure 2:**
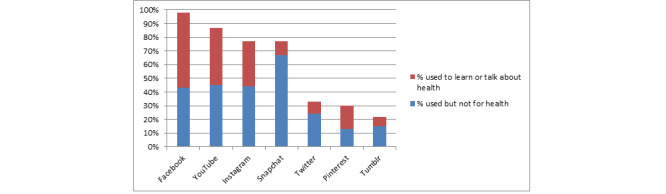
Proportion using different social media channels, including those who used to talk about health (N=165).

In total, 89.1% (147/165) of the participants said they used social media 3 or more times a day; 10.9%, (18/165) twice a day. Facebook, YouTube, Snapchat, and Instagram were all used by most participants and Twitter, Pinterest, and Tumblr, by some (Figure 2). Overall, 67.8% (112/165) used social media to talk about or learn about health. Among users, approximately half used Facebook, YouTube, Instagram, and Pinterest for health reasons, but some of the users used Snapchat, Twitter, or Tumblr for health purposes.

In polls, participants generally agreed that they could easily find information on the web to help them be healthy, with a median score of 6 out of 7 (IQR 5-6). They also reported a fairly high level of trust in the information they found on the internet, with a median score of 5 out of 7 (IQR 4-6).

**Table 1:** Participant demographics (N=165).

Characteristics		Values, n (%)
**Gender**		
	Female	99 (60)
	Male	65 (39.4)
	Nonbinary or gender queer	1 (0.6)
**Age (years)**		
	18-21	91 (55.2)
	22-24	74 (44.8)
**Language spoken at home**		
	English	121 (73.3)
	Other	44 (26.7)
**Currently studying**		
	Yes	110 (66.7)
	No	55 (33.3)
**BMI (kg/m^2^)^a^**		
	Underweight (<18.5)	17 (10.3)
	Healthy weight (18.5-25)	88 (53.3)
	Overweight (25-30)	36 (21.8)
	Obese (>30)	24 (14.5)
**Live with parents**		
	Yes	81 (49.1)
	No	84 (50.9)
**Location of residence**		
	Metropolitan	135 (81.8)
	Rural	30 (18.2)

^a^BMI by self-reported weight and height, with World Health Organization cut-offs applied.

### Use of Social Media Channels

#### Social Media Channels

Different channels were used differently for health purposes. Even when participants described following the same companies or individuals across multiple platforms so they could see all their available content, they tended to use each platform differently:

Since last year I’ve been following [influencer – name redacted]. I discovered her via Pinterest and then subscribed to her email list in which I get sent monthly workout calendars for each day of the month that are linked to her online youtube channel that shows you the workout. I also access her website for healthy recipes and nutritional infographics relating to clean eating, antioxidants and superfoods, etc.

Female participant, aged 18-21 years, low health interest

#### Facebook (Social and Scrolling)

Many young adults described Facebook as a boredom killer. This meant they spent a lot of time scrolling through their newsfeeds, which often resulted in seeing health-related content. These were often in the form of news articles that appeared on their newsfeed or advertisements and sponsored posts, particularly from health-related retail companies. Many participants described tuning out or not paying attention to advertisements on their newsfeeds; some people believed they naturally do not notice advertisements as they are used to ignoring them. Clickbait was discussed in both positive (attention-grabbing, making them want to read the information) and negative ways (reduces the credibility of the information source and decreases the likelihood that they will click on the source, as it would likely only have sensationalized information):

Facebook: I only use it when I’m bored/to message friends, so about two hours of usage a day.

Male participant, aged 22-24 years, high health interest

I tend not to seek out information about having a healthy lifestyle, but I do occasionally click on articles on my facebook feed about health.

Female participant, aged 18-21 years, high health interest

I noticed a facebook ad for skill share, my local gyms, the digital garage training by google and Specsavers. I noticed them as I scrolled through my facebook feed because you sometimes forget that there are ads within facebook and assume that every post is from something that you have “liked.”

Female participant, aged 18-21 years, high health interest

On the other hand, many actively sought out information about specific health areas they were interested in, usually through joining groups or following relevant pages. A few also mentioned finding relevant local classes and events through Facebook. Young adults were able to find groups that aligned with their specific interests and outlook, including those that have shared similar life experiences to them, such as having the same medical condition. These groups were viewed highly and often were used as the first point of call for information related to many health issues. The participants did not often discuss how they found these pages or why they specifically chose to follow those pages or groups that they did:

[Magazine - name redacted] have a Facebook page where they post articles about what are the right exercises to do for a specific muscle, reading it helps me understand my body much more easily.

Male participant, aged 18-21 years, high health interest

Oh god, SO many facebook groups! I’ve started following a lot of recipe pages and I’m in a great group called [Facebook group - name redacted] that's very anti “health-veganism”. That’s not to say that people don’t share awesome healthy recipes that they’ve created, but more that it’s not about health-shaming or policing what people eat, more about helping them to lead happy lives eating in a way they feel is ethically sound. it's great because so many vegan pages are really elitist, and this one makes a point that veganism can be fun and delicious and interesting without being judgemental or condescending.

Nonbinary gender participant, aged 22-24 years, high health interest

For discussion/forums, if it a personal issue I will then search for a FB group to join. You definitely learn a lot more from others personal experiences with a medical issue.

Female participant, aged 22-24 years, high health interest

The participants also described health-related interactions with their friends through Facebook. For example, they described being influenced by friends sharing information about their health behaviors and lifestyle. They also witnessed or engaged in discussions among friends about health topics, such as the gym and healthy eating, either publicly on Facebook or in Facebook private messenger groups. People noted that friends shared positive stories about successful weight loss:

Got persuaded to get healthier through facebook where friends posted their workout routines and summer bodies haha, it got me thinking how i would feel if i workout and get better too.

Male participant, aged 22-24 years, low health interest

Friends also communicated with each other by sharing or tagging advertising content. Some participants would base their willingness to buy health products that companies were selling on their friends’ experiences or experiences and reviews from other people. They would often trust these products if they received positive feedback:

My friendship circle basically communicates via sharing, tagging and liking each other in these posts...for example my bestie is obsessed with the latest [supermarket - name redacted] ad with the pasta sauces and will message me when she sees it usually just saying “PASTA AD” it’s hilarious.

Female participant, aged 18-21 years, low interest in health

I noticed some of my friend’s discussing brands of protein powders and whether or not they are really effective or just a money-grabber which I thought was interesting.

Female participant, aged 18-21 years, low health interest

Buzzfeed, particularly the Tasty channel, was mentioned by many young adults who were predominantly exposed to this channel through Facebook. It was praised for showing delicious food and easy to follow recipe videos and tutorials. The content was described as high quality and informative:

Food videos, sites like Tasty and Buzzfeed Food are amazing! The food always looks so good, and they provide really simple recipes for people to try! I have so many in my saves for later! I always share those two things around, I feel like they bring a happiness or a joy to my feed that other people can enjoy and get a kick out of as well!

Female participant, aged 18-21 years, high health interest

Some participants stated that information on Facebook was unreliable or misleading because the information could be from anyone and does not necessarily reflect the opinions of health professionals. Information clearly provided by a health professional was considered trustworthy. Other participants described the information on Facebook as unreliable but with no specific reason as to why.

#### YouTube (Instruction Kit for Life)

YouTube was considered a useful source for learning about everything and was often the go-to when searching for information or advice (after Google). YouTube was something they used regularly either directly as a search engine or more passively through videos recommended by YouTube based on their previous search history. Many liked the video format that was easier to follow than reading the text. Demonstrations and tutorials in visual format also made it easier to learn new skills and check in that they were doing things properly. Work out videos were particularly popular and were cited as a quick and free alternative to gyms:

I get most of my health content from youtube, usually because an actual demonstration of how to do an exercise or how to cook something is really helpful, and it makes me more motivated to follow through.

Female participant, aged 22-24 years, high health interest

I really just get most of my information about health and fitness from youtube. I am one of those people who can’t really learn that well by just reading off a web page. I would much rather watch a video about someone doing something, I learn a lot more that way.

Male participant, aged 22-24 years, high health interest

A small number of participants sought advice on specific health-related issues from health professionals on YouTube:

For any physiotherapy related information, I watch the video on the [channel - name redacted]. They have over 1000 videos explaining exercises to combat any muscle related injury. On a side note, I was suffering from a knee clicking issue last month and following this exercise [link] helped me overcome that issue.

Female participant, aged 18-21 years, high health interest

Young adults also found inspiration from people they had seen sharing their stories on YouTube. The participants liked to learn what had worked for others and what could work for them:

I follow a few youtubers who post vlogs and they sometimes include “what I ate in a day” or “my fitness routine” which does inspire and motivate me to maintain my healthy lifestyle.

Female participant, aged 18-21 years, high health interest

Or I’ll look up blogs on YouTube to see what has worked for individual people and what they think about specific lifestyles, food recipes, weight loss methods, alternative health methods etc.

Female participant, aged 18-21 years, high health interest

These YouTubers were seen as open and honest, they shared large volumes of information about their diet and exercise, and provided tips and advice based on their personal experience. YouTube was seen as more personal than some of the other formats often described because they were *real people* that you could see through their videos. Trustworthiness was often based on how a YouTuber looked, their personality, likeability, first impressions, and what they perceived of the person. Videos with high production value that looked professional and included a real person talking about the topic were considered more trustworthy compared with a robotic computer-generated voice and stock images:

I tend to scroll past all the “fitspo” looking bloggers - i don’t have a lot of trust in them because often they are also incorporating surgical enhancements to achieve their physiques, and also are often pushing particular products, or just lack real knowledge. I came across [influencer - name redacted] videos, including this one: [link] I trusted her more because she didn’t look “plastic” -she came across as someone from the industry who is knowledgeable about fitness, rather than someone who got into it because it is cool.

Female participant, aged 22-24 years, high health interest

The “bullshit alarm” rings when the voiceover is done by a robotic voice and stock Google Images are used. I would be sure to double check the facts.

Male participant, aged 18-21 years, low health interest

To gather reliable information, some described watching multiple videos to cross-check their facts. Some people described that they could not always find the information they needed on YouTube and might try a subsequent source such as Google. Participants would sometimes look at YouTubers with lots of subscribers or videos with lots of views as trustworthy. Fake sounding or overdramatic information and the inclusion of a large amount of apparent product sponsorships were also seen as untrustworthy. Many participants felt that they could just instinctively determine whether the information was correct:

Things that make me trust what I see is stories of other people that have used that information and it working for them and statistics are a good way to get my attention. I like the website WebMD and youtube as a way to visually see the effects it has on people e.g. diets. What I find frustrating is when you’re searching for answers to your problems but all the company is doing is promoting themselves.

Female participant, aged 18-21 years, low health interest

I would begin by looking through you tube and trying to find a few sources that look credible. I would look at the length of the video, the amount of views it has and the amount of likes to dislikes ratio in order to gauge the reliability of the source...I would then cross check the information between different videos in order to gauge how true the information is. If the information is outlandish or just does not seem correct then I would continue further research.

Male participant, aged 22-24 years, high health interest

Many participants described tuning out or not paying attention to advertisements at the beginning of YouTube videos; some people believed they naturally do not notice advertisements as they are used to ignoring them. Using applications that block advertisements, such as *adblocker*, was also very common.

#### Instagram (Inspirational Appearances)

Instagram was primarily described as a source of inspiration for health and wellness. Particularly inspiring were those whose lives were dedicated to healthy lifestyles, nutrition, and fitness, including celebrities, influencers, models, and personal trainers:

I follow some models and yogis who generally inspire me to practice more and eat more acai bowls.

Female participant, aged 18-21 years, low health interest

The focus was solely on appearance. For food, clean eating and *food porn* were frequently mentioned, and concepts such as clean eating were equated with health. Most of those discussing using Instagram for health information were women, who followed other women and aspired to be like them or sought to emulate aspects of their idealized lifestyles. There was a clear emphasis placed on the ideal attractive female body, where a *good body* was equated to health:

If I go on Instagram, I often see fit and pretty models that make me want to start being healthy.

Female participant, aged 18-21 years, high health interest

I continue to visit this model’s Instagram page because I trust the information she provides, since it is her job to look her best all the time and many of her posts are not sponsored but rather candid snippets of her lifestyle.

Female participant, aged 18-21 years, high health interest

Advertising was also prevalent on this platform, including both overt advertising and the promotion of products by Instagram influencers or celebrities. Advertising content was often hidden among Instagram posts on their feeds and was not always distinct from other content:

Also checked instagram a few times i have noticed a lot of celebrities or “instagram famous” people advertising all different products including weightloss shakes and teas although it isn't exactly an ad it is its clear that they have been paid to post about it.

Female participant, aged 22-24 years, low health interest

On instagram I scroll top to bottom, and get confused when a post shows up from someone I don’t follow: until I realise its an ad. More likely to actually watch videos or something that tricks me into thinking it belongs on my timeline if that makes sense: if its clearly out of place, I’m more likely to ignore completely.

Female participant, aged 18-21 years, low health interest

Instagram was often mentioned among other web-based and social media sources, mostly not as an individual’s only source or main source of health-related information.

#### Pinterest (Curating Healthy Ideas)

A smaller group used Pinterest for a range of health reasons, but they were quite enthusiastic about it. Pinterest was used for inspiration for new ideas and tips, especially recipes and workouts. People also shared their own content to get involved in conversations. Visual presentations, including photos and infographics, were considered appealing and engaging. Pinterest was mainly used as a search engine and was useful because of its ability to create *boards* to save information and ideas for use later:

I do also look at health content on Pinterest - being so visual it is really engaging, and when it comes to recipes, a gorgeous photo makes me so much more likely to actually cook the meal! Pinterest also has a large number of people who are interested in meal prep, so it’s easy to find inspiration.

Female participant, aged 22-24 years, high health interest

I love looking at Pinterest and usually save on my page detox diets health routines and fitness workouts on my page. I find it quite interesting and fun because they seem so simple on pinterest with their diagrams and quick and easy recipes. I think their so convienent.

Female participant, aged 22-24 years, high health interest

#### Twitter, Tumblr, and Snapchat (Not About Health)

These platforms were used by young adults but rarely for sourcing or discussing health information. A few participants mentioned following health-related personalities or companies such as *Taste* or *The Food Network* on Snapchat. Otherwise, Snapchat was used to chat with friends; Twitter was used to follow news, celebrities, and sports; and Tumblr, for memes.

## Discussion

### Principal Findings

Young people in this study reported that they easily found health information on the web and frequently used Facebook, YouTube, and Instagram for sourcing health information. These platforms were used differently: Facebook was used to scroll through and kill time, YouTube was used to deliberately search for advice and instructions, and Instagram was used as a source of inspiration. Health information was usually found by accident on Facebook, through advertisements or friends or by joining groups or following pages related to specific health topics of personal interest. On YouTube, health content was searched for deliberately, with multiple videos being checked to compare information given; in many cases, the quality of the information was judged based on the perceived trustworthiness of the YouTuber personality presenting it. Instagram-based health information was driven almost completely by fitness and wellness personalities. Previous health promotion using social media has relied on information dissemination and social support [1]; however, the young people in our study were more interested in using social media for inspiration and operational instruction.

### Trustworthiness of Health Information on Social Media

The rise of social media as an information source has resulted in a largely unregulated body of information related to health and nutrition, making it difficult to determine what information is evidence-based [24]. Participants indicated looking to social media influencers for health and lifestyle inspiration despite these individuals often promoting information that lacks an evidence base [23,25]. The often unrealistic lifestyles promoted by these influencers and social media *fitspiration* content has potentially damaging effects on mental health and well-being, body image, and food choices [34,35]. There is a need for the public to develop media literacy skills to decipher credible sources and messages, particularly in response to the changing and evolutionary nature of a science such as nutrition and to potentially build resilience from the damaging aspects of social media [36,37].

Participants reported a fairly high level of trust in health information on the web; however, they largely relied on their instinct to determine whether information was trustworthy. Trust was predominantly based on the trustworthiness of the person and the quality of the presentation rather than on the basis of the platform or the content of the information. An individual’s trustworthiness was based on their appearance and perceived *realness*. Realness was based on sharing personal stories and personal experience; for example, if someone shared that something had *worked for them personally*, it was considered evidence of effectiveness. This builds on existing media research demonstrating that the trustworthiness of the person communicating information is the most important factor in determining the trustworthiness of a message [38] and that this is not correlated with the expertise of the source [39]. The perceived credibility of different sources on social media is influenced by factors such as the type of language used, specifically the use of positively framed messages and nonbiased or opinionated messages [27]. Also affecting credibility were bandwagon heuristics such as the number of followers or likes someone on social media has and expertise heuristics such as relation to a well-known credible organization [27]. The factors that young people use to judge credibility and source authority are not consistent with recommendations; these generally suggest using or citing professionals and not testimonial style personal stories (eg, Better Health Channel [40]). Health promoters should attempt to create higher levels of personal trust in our messages, for example, by having real researchers, practitioners, or young adult ambassadors present information directly to young people and building a relationship with their audience. An experimental study also showed that nutrition professionals can enhance their trustworthiness and authenticity by using *heroic* messaging featuring positive emotions [27]. Furthermore, health experts can work with existing influencers to share evidence-based health information with their followers. There is some evidence that this can increase the reach and engagement of health campaigns [41].

### Platforms for Health Information

This research also shows that health promotion campaigns need to be adapted to suit different platforms. Each platform has a different style of content and method of finding content. Health promotion efforts may benefit from using platforms in the way that young people already use them; for example, using role models on Instagram and YouTube, eye-catching headlines or support groups on Facebook, and detailed but easy to follow instructions via YouTube. We should provide messages that resonate with and use the language of our target audience. Messages should be simple and easy to understand, including a call-to-action that is achievable and communicates a tangible benefit, such as easy recipes or workouts [42]. We recommend that time should be invested in analyzing *competitor* posts to gain insights into strategies that are engaging with the target audience [17,21]. Science translation needs to be memorable, easy to visualize, and should specify when and how to act on the scientific recommendation to increase understanding and uptake of behaviors that the messages promote, by the intended target audience [43]. Overall, using the concept of real, relatable people sharing anecdotes backed by evidence is likely to be a valuable strategy for social media health communication.

Another key finding is that YouTube is likely underutilized by health promoters. After Facebook, YouTube was the second most used platform overall and for health information by young people, but it has been relatively little studied in terms of health promotion research. Although past research mentioned YouTube as being used as a time waster [13], we found that it was also used very deliberately to seek health information and most importantly, practical instructions and guidance.

It is difficult to attract young people to a profile without a pre-existing set of followers or access to a follower group. Paid advertising is a route often used by health promotion and research to reach young people. Although many participants said that they were wary of any sponsored content, their comments revealed that they often did notice and engage with them and that advertisements were cleverly placed to be confused with natural content. Many stated that they were immune to advertising, that they just did not see it, but research shows that this sort of covert advertising on social media can be very effective [44,45]. The use of advertisement blocking software by young adults was common and may affect the reach of health promotion or social marketing campaigns that target people on social media, as these campaigns will show up as sponsored posts or advertisements unless the individuals being targeted follow the organization’s page.

### Limitations

The key limitation of this study is that respondents generally did not provide in-depth responses to prompts as would be possible in a traditional qualitative interview. There was some back-and-forth discussion and requests to expand on comments from moderators and other participants, but extensive probing and follow-up on comments was not possible. We would recommend that future studies using this methodology include moderators who are age-group peers of participants to increase their engagement.

### Conclusions

This study provides insight into the different ways young adults use social media platforms to source health information. Health experts and health promotion practitioners could improve their engagement with young adults by using relatable and inspiring personalities, particularly on YouTube and Instagram. As new social media platforms evolve and their popularity waxes and wanes, health professionals should be cognizant of the platform used by their target audience and the types of messages that promote high engagement.
